# Lotus leaf extract inhibits ER^−^ breast cancer cell migration and metastasis

**DOI:** 10.1186/s12986-021-00549-0

**Published:** 2021-02-18

**Authors:** Yuelin Tong, Zhongwei Li, Yikuan Wu, Shenglong Zhu, Keke Lu, Zhao He

**Affiliations:** 1 Department of Endocrinology, Shandong Provincial Hospital, Cheeloo College of Medicine, Shandong University, Jinan, Shandong China; 2 State Key Laboratory of Food Science and Technology, School of Food Science and Technology, Jiangnan University, Wuxi, 214122 China; 3 Shandong Key Laboratory of Endocrinology and Lipid Metabolism, Jinan, China; 4 Shandong Provincial Hospital Affiliated to Shandong University, Jinan, China

**Keywords:** Lotus leaf, Cell migration, Breast cancer, Metastasis, TGF-β1 signaling pathway

## Abstract

**Background:**

Patients with estrogen receptor negative (ER^−^) breast cancer have poor prognosis due to high rates of metastasis. However, there is no effective treatment and drugs for ER^−^ breast cancer metastasis. Our purpose of this study was to evaluate the effect of lotus leaf alcohol extract (LAE) on the cell migration and metastasis of ER^−^ breast cancer.

**Methods:**

The anti-migratory effect of LAE were analyzed in ER^−^ breast cancer cells including SK-BR-3, MDA-MB-231 and HCC1806 cell lines. Cell viability assay, wound-healing assay, RNA-sequence analysis and immunoblotting assay were used to evaluate the cytotoxicity and anti-migratory effect of LAE. To further investigate the inhibitory effect of LAE on metastasis in vivo, subcutaneous xenograft and intravenous injection nude mice models were established. Lung and liver tissues were analyzed by the hematoxylin and eosin staining and immunoblotting assay.

**Results:**

We found that lotus LAE, not nuciferine, inhibited cell migration significantly in SK-BR-3, MDA-MB-231 and HCC1806 breast cancer cells, and did not affect viability of breast cancer cells. The anti-migratory effect of LAE was dependent on TGF-β1 signaling, while independent of Wnt signaling and autophagy influx. Intracellular H_2_O_2_ was involved in the TGF-β1-related inhibition of cell migration. LAE inhibited significantly the breast cancer cells metastasis in mice models. RNA-sequence analysis showed that extracellular matrix signaling pathways are associated with LAE-suppressed cell migration.

**Conclusions:**

Our findings demonstrated that lotus leaf alcohol extract inhibits the cell migration and metastasis of ER^−^ breast cancer, at least in part, via TGF-β1/Erk1/2 and TGF-β1/SMAD3 signaling pathways, which provides a potential therapeutic strategy for ER^−^ breast cancer.

## Introduction

Breast cancer is usually classified into three subtypes according to the expression of hormone receptors: estrogen receptor positive/progesterone receptor positive (ER+/PR+), human epidermal growth factor receptor positive (HER2+) and triple negative (TNBC) [1]. Among the three subtypes of breast cancer cells, ER^−^ breast cancer such as HER2+ and Triple negative breast cancer cells have a higher migration capacity than that of ER+/PR+ breast cancer cells. In breast cancer patients, HER2+ subtypes of breast cancer accounts for 20–30%, which is an invasive tumor with shorter metastatic time and poor prognosis compared to other two subtypes of breast cancer patients [2]. In addition, Triple negative breast cancer (TNBC) is considered as the most severe subtypes of breast cancer because of high rate of recurrence and metastasis [3]. However, there is no effective treatment and drugs for ER^−^ breast cancer metastasis.

About 90% death rates of breast cancer resulted from metastasis. Cell migration is a complicated process associated with NF-κB, TGF-β1 and Wnt/β-catenin signaling pathways [4–6]. TGF-β1 signaling pathway is a pivotal pathway for cell migration in breast cancer. TGF-β enhances ROS levels by enhancing production and reducing antioxidative/scavenging systems activity [7, 8]. And enhanced ROS levels in turn may promote the TGF-β expression and release from the secreted complex in cell [9]. Moreover, several studies have demonstrated that the ROS level is associated with carcinogenesis, abnormal growth, and angiogenesis, especially metastasis [10–14].

Lotus leaf (*Nelumbo nucifera* Gaertn) is a traditional Chinese medicine, also called He-Ye, which has a long history of usages against oxidation, diabetes, obesity and immunomodulatory effects in China [15]. Considerable literatures have demonstrated that lotus leaf contains nuciferine, quercetin, quercetin-3-O-glycoside, kampherol-3-O-glycoside, and myricetin-3-O-glucoside [16]. These compounds from lotus leaves, seed and rhizome display cytoprotective, anti-bacterial, anti-obesity especially anti-oxidant pharmacologic activities [17–21]. However, the effect of lotus leaf extract on cancer metastasis still remained unclear.

In this study, our data showed that LAE significantly inhibits the ER^−^ breast cancer cell migration and metastasis via the SMAD3 and Erk1/2 signals, accompanied by reduced intracellular H_2_O_2_ level in ER^−^ breast cancer cells. RNA-sequence analysis also showed that extracellular matrix signaling pathways and FAK might be another possible signaling pathways that are involved in the inhibition of cell migration induced by LAE. These results suggested that LAE has the potential to figure out novel components to develop drugs for the treatment of ER^−^ breast cancer.

## Materials and methods

### Cells culture

Breast cancer cell lines SK-BR-3, HCC1806 and MDA-MB-231 were purchased from cell bank of China. Cells were maintained in RPMI 1640 (HCC1806) and DMEM (SK-BR-3 and MDA-MB-231) media (Gibco, USA) with 10% FBS (Gibco, USA) and 100 µg/ml penicillin/streptomycin (Gibco, USA) in a humidified atmosphere containing 5% CO_2_ at 37 °C.

### LAE treament

The dry lotus leaf (*Nelumbo nucifera* Gaertn, http://www.theplantlist.org) powder was purchased from Tong Ren Tang Group in Wuxi, China, in November 2017 (Latitude: 31° 33′ 17.44″ N; Longitude:120° 19′ 15.24″ E). The company is approved by the competent department, ensuring the good production and quality according to the approved content. All production lines had passed the national GMP recognition. Then, the lotus leaf was identified in National Functional Food Engineering Technology Research Center in Jiangnan University (China).

Lotus leaf alcohol extract: The lotus leaf powder and the 75% aqueous ethanol solution were uniformly mixed at a ratio of 1:20, and shaken at 37 °C for 12 h. Then separated by filtration, and the obtained residue was repeatedly extracted twice. All the collected filtrate was concentrated to dryness by evaporation on a rotary evaporator, and then dissolved in DMSO and filtered by a membrane filter with 0.22 μm pore size (Millipore, USA). Finally, LAE was diluted into several concentrations (10, 25, 50, 100, 250 µg/ml) for cell treatments and stored at 4 °C.

### Human breast cancer xenograft model

This study was approved by the ethics committee of Jiangnan University with protocol number SYXK2016-0045. Female BALB/c nude mice, 4 weeks of age, were purchased from Lingchang Biotechnology Co., Ltd. (Shanghai, China). All the experiments were in accordance with national institution guidelines. MDA-MB-231 cells (1 × 10^6^) suspending in medium were subcutaneously injected in the right flank of mice. After 1 week of injection, the mice bearing tumor were randomly subdivided into 2 groups and administered via diet either with vehicle (model group) or 0.5% (w/w diet) LAE. Tumor dimensions were measured with vernier caliper and tumor volumes were estimated by the formula: length × width^2 × 0.5. Body weights and tumor growth were recorded twice a week. The mice were sacrificed after 56 days according to the appearance of metastasis, and visible metastatic tumors were counted and subjected to further analysis.

### Tail vein injection model

This study was approved by the ethics committee of Jiangnan University with protocol number SYXK2016-0045. Female BALB/c nude mice, 4 weeks of age, were purchased from Lingchang Biotechnology Co., Ltd. (Shanghai, China). All the experiments were in accordance with institution guidelines. MDA-MB-231 cells (5 × 10^5^) suspending in PBS were injected through the tail vein of mice. Then, all the mice were randomly subdivided into 2 groups (10 mice/group) and administered via diet either with vehicle (model group) or 0.5% (w/w diet) LAE in the diet. Body weights were scaled once a week. The mice were sacrificed after 41 days according to the appearance of metastasis, and the metastatic tumors and the organs with visible metastatic tumors were analyzed.

### Cell viability assay

Cell Counting Kit 8 (CCK8) was purchased from Dojindo Molecular Technology (Tokyo, Japan). Cells were cultured in 96-well plates at a density of 5000 cells per well in 100 μl medium. LAE were added into the wells and incubated for 48 h. Then, cells were added 10 μl CCK8 substrate and incubated for another 2 h at 37 °C. The optical density was measured at 450 nm on a microplate reader Multiskan GO (Thermo Scientific, USA).

### Wound-healing assay

Cells were seeded in dish to form a confluent monolayer. Then 200 μl pipette tip was used to make a scratch in the middle of plate, and wash twice with PBS. The monolayer was maintained with the FBS-free media with LAE supplement. Wound closure was monitored after 0 and 36 h. The percent of wound closed are calculated by image J. TGF-β1 was purchased from Peprotech (USA, # 100-21-10) and dissolved in 10 mM citric acid, pH3.0 at 0.1–1.0 mg/ml. Chloroquine diphosphate (CQ) was purchased from Medchem Express (Shanghai, China, cat# HY-17589) and dissolved in H_2_O to 10 mg/ml. LiCl was purchased from Sigma-Aldrich (Sigma, St. Louis, MO, USA, # 213233) and dissolved in cell culture medium to 20 mM. Nuciferine was purchased from Medchem Express (Shanghai, China, cat# HY-N0049) and dissolved in DMSO to 1.6925 mM.

### H_2_O_2_ content

The content of H_2_O_2_ was measured by the Hydrogen Peroxide assay kit (Beyotime, # S0038) according to the manufacturer protocols. In brief, test tubes containing 50 μl test solutions were placed at room temperature for 30 min and measured immediately with a spectrophotometer at a wavelength of 560 nm. Absorbance values were calibrated with a standard curve generated with known concentration of H_2_O_2_.

### Cell signaling

Cells were lysed by lysis buffer (RIPA buffer contains protease inhibitors and phosphatase inhibitors). Protein concentrations were determined by using a BCA Protein Assay Kit. Equal amounts of protein were separated in 10% SDS–polyacrylamide gels, and then transferred onto PVDF membranes (Millipore, Beijing, China). The membranes were blocked with 5% fat free milk for 1 h at room temperature, further incubated with primary antibodies (1:1000) and then probed with secondary peroxidase-labeled antibody. The signal was detected by Plus-enhanced chemiluminiscence using FluorChem FC3 (ProteinSimple, USA). The following primary antibodies were used: Erk1/2 (# 9102), p-Erk1/2 (# 4370), SMAD3 (# 9523S), TGF-β1 (# 3711S), Snail (# 3879T), PTEN (# 9552S), β-catenin (# 8480S), p-AKT (# 4060), AKT (# 4685), p-JNK (# 9251S), JNK (# 9252T), p-p38 (# 4511), p38 (# 8690), p62 (# 23214S), LC3 (# 3868T), Catalase (# 12980T) were purchased from cell signaling technology; NOX4 (# ab133303), p-SMAD3 (# ab52903) and NOX2 (# ab129068) were from Abcam company; β-Actin (# sc-130656) and SOD1 (# sc-101523) were purchased from Santa Cruz Biotechnology.

### Hematoxylin and eosin staining

For histopathology analysis, lung and liver tissues were fixed by paraformaldehyde and then were dehydrated, embedded in paraffin and cut into serial sections at 5 μm. Sections were stained with hematoxylin and eosin (H&E) solution and observed them under an optical microscope (DP73, OLUMPUS, Japan).

### Statistical analysis

All experiments were performed at least three times and data were presented as mean ± SEM. One-way ANOVA with Dunnett’s post-test was performed (*P < 0.05; **P < 0.01; ***P < 0.001).

## Results

### LAE inhibits ER^−^ breast cancer cells migration independent of nuciferine

**Fig. 1:**
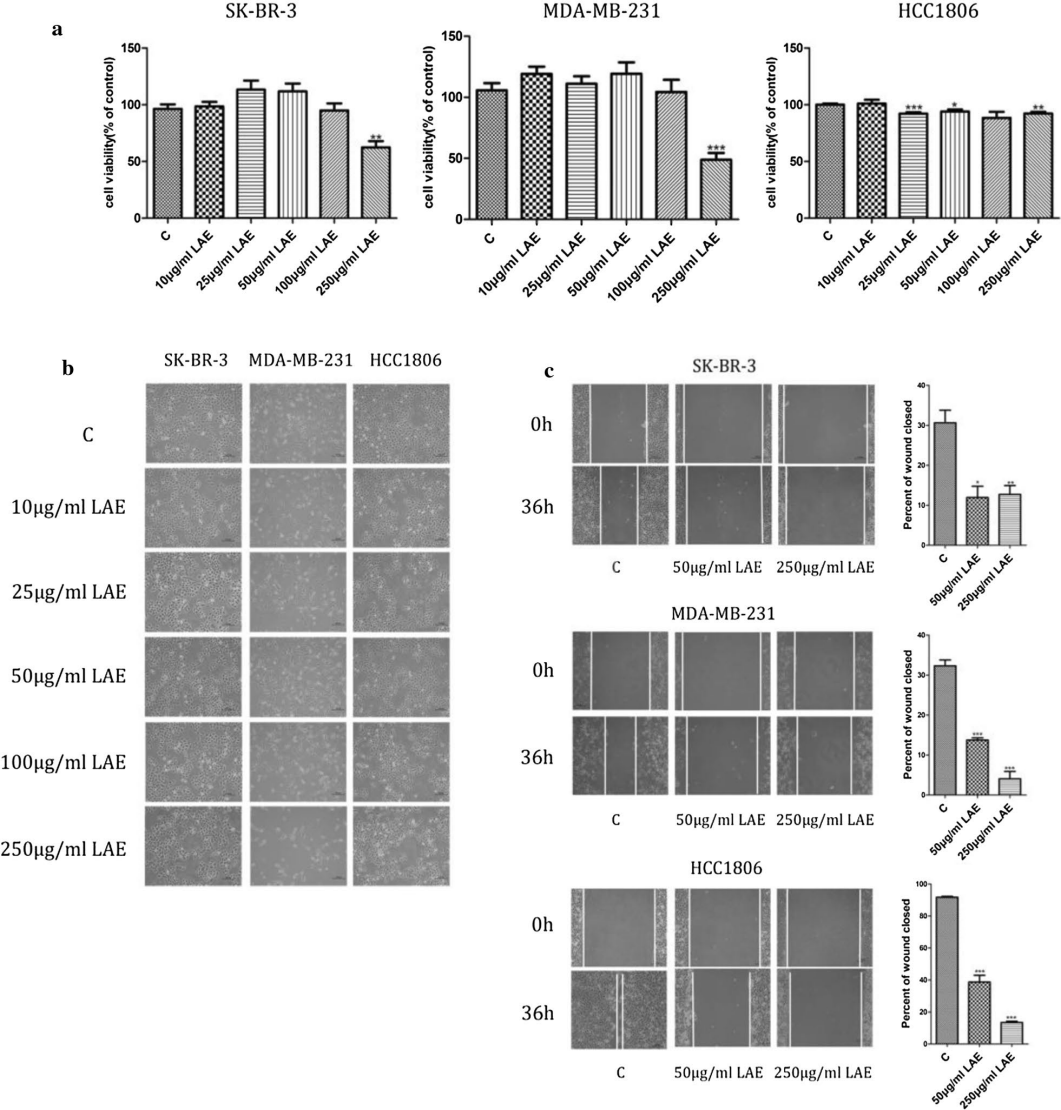
LAE effect on ER^−^ breast cancer cells migration. SK-BR-3, MDA-MB-231 and HCC1806 breast cancer cells were treated with different concentrations of LAE for 48 h. **a** The viability of LAE-treated cells was determined by CCK8 assays. **b** The cell morphology after treating with LAE. **c** Wound healing assays after treating with LAE for 36 h (× 100 magnification)

To investigate LAE effects on cell migration, SK-BR-3, MDA-MB-231 and HCC1806 ER^−^ breast cancer cell lines were used in the study. Previous studies have shown that cell proliferation interferes with cell migration. To rule out the effect of cell growth, the proliferation of breast cancer cells was examined by CCK8 assay with LAE supplement. The viability of SK-BR-3, MDA-MB-231 and HCC1806 cells were similar between with low dose (50 and 100 µg/ml) of LAE supplement and without LAE (Fig. 1a). All of SK-BR-3, MDA-MB-231 and HCC1806 cells exhibited unchanged cell morphology with 100 µg/ml or less LAE supplement (Fig. 1b). However, cell number was significantly reduced with high doss (250 µg/ml) of LAE supplement for 48 h (Fig. 1a). These results indicated that low dose LAE did not alter breast cancer cell proliferation.

Next, we examined the LAE role in cell migration by wound healing assays with 50 and 250 µg/ml LAE supplement for 36 h. Previous studies found that FBS promote cell proliferation to alter cell migration. To rule out the effect of FBS, all experiments were performed in an FBS-free medium. Cell wound healing distance analysis showed that LAE supplement significantly reduced the wound healing distance in three subtypes cells, suggesting cell migration was suppressed (Fig. 1c). Interestingly, 50 or 250 µg/ml LAE supplement showed similarly inhibitory effect on SK-BR-3 cells migration, while LAE supplement inhibited cell migration of MDA-MB-231 and HCC1806 cells in a dose dependent manner.

Nuciferine, a bioactive component of lotus, inhibits the growth of cancer cells and breast cancer-associated bone loss [22, 23]. To verify if nuciferine is involved in the inhibition of cell migration by LAE, SK-BR-3, MDA-MB-231 and HCC1806 cells were treated with nuciferine supplement. Cell viability (Additional file 1: Fig. S3a) and migration (Additional file 1: Fig. S3b) were similar in the presence or absence of high dose of nuciferine, indicating that nuciferine was not associated with LAE-inhibited cell migration. Together, these results indicate that LAE supplement suppresses ER^−^ breast cancer cells migration.

### LAE-inhibited cell migration is independent of autophagy and Wnt signals

Autophagic process is recognized to suppress cancer metastasis through decreasing EMT [24]. To determine if LAE-suppressed cell migration was regulated by autophagy and EMT-related Wnt/β-catenin signaling pathway directly, we examined β-catenin, snail, p62 and LC3 proteins levels by immunoblotting. Elevated LC3 protein level was observed in cells with high dose (100, 250 µg/ml) of LAE supplement. However, p62 protein level was similar in cells with low and high dose of LAE supplement (Additional file 1: Fig. S1a). To verify the controversial results, we used autophagy/lysosome inhibitor chloroquine (CQ) to inhibit autophagy flux. Inhibition of autophagy flux did not alter cell migration, revealed by wound healing distance (Additional file 1: Fig. S1b). Unchanged β-catenin and snail protein levels were observed in three subtypes of breast cancer cells with low or high dose of LAE supplement (Additional file 1: Fig. S1c). To further determine the role of Wnt signaling pathway on the effect of LAE-inhibited cell migration, LiCl was used to activate the Wnt signal. Wound healing assays revealed that the activation of Wnt signal did not reverse the cell migration (Additional file 1: Fig. S1d), suggesting LAE-suppressed cell migration was independent of Wnt signaling pathway. Thus, LAE-suppressed cell migration is independent of autophagy and Wnt signaling pathways.

### TGF-β1 is associated with LAE-suppressed cell migration

**Fig. 2:**
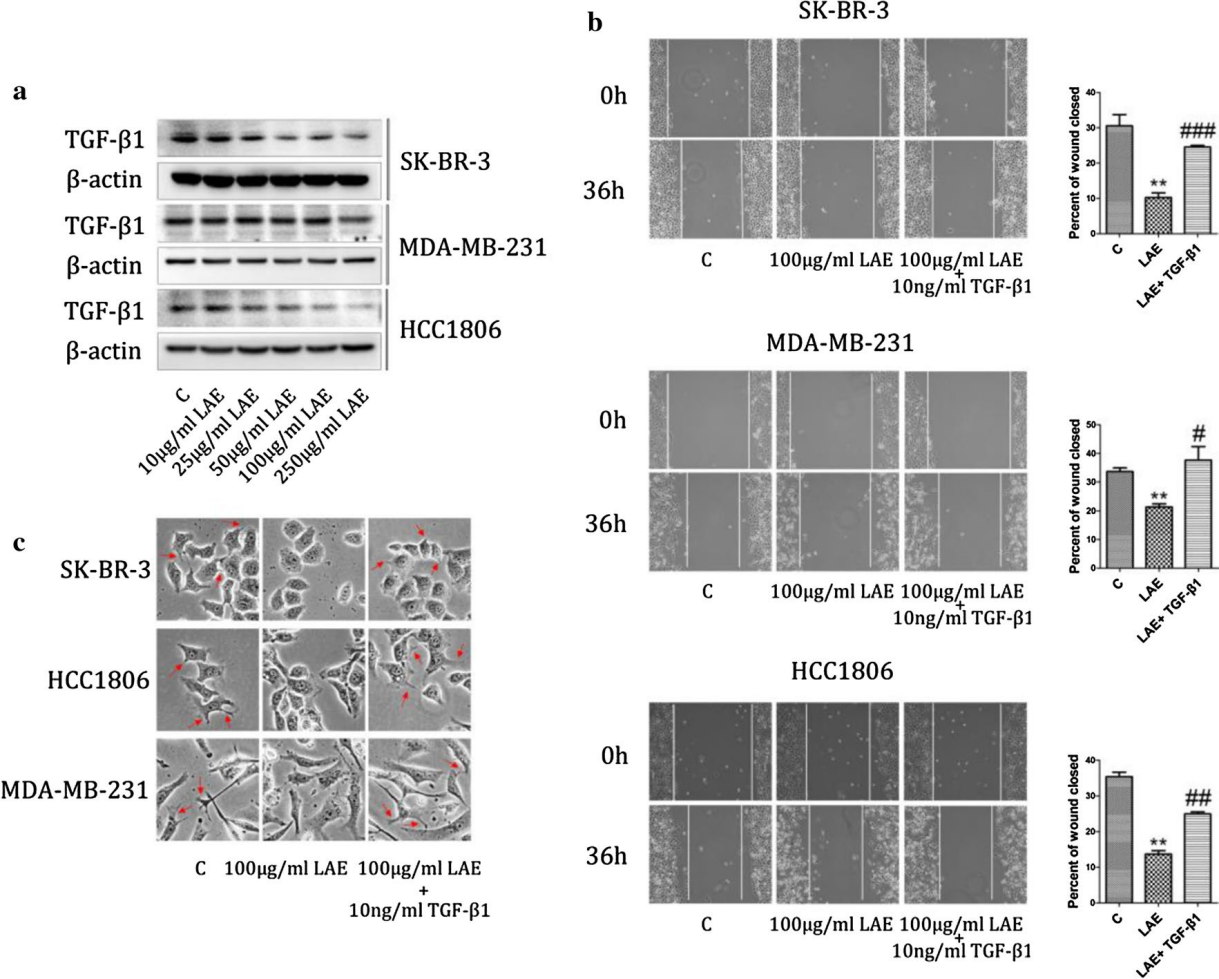
TGF-β1 signal with LAE supplement**. a** TGF-β1 was determined by immunoblotting under different concentrations of LAE (0, 10, 25, 50, 100, 250 µg/ml). **b** Wound healing assays after treating with 100 µg/ml LAE alone or combined with 10 ng/ml TGF-β1 for 36 h (× 100 magnification). **c** The morphology of SK-BR-3, MDA-MB-231 and HCC1806 breast cancer cells after exposure to 100 µg/ml LAE alone or combined with TGF-β1 (10 ng/ml) for 48 h (× 400 magnification)

Considerable literatures showed that TGF-β1 is recognized as a major regulator of EMT to regulate tumor metastasis [25–27]. Then we tested the protein level of TGF-β1 after treating with LAE. The results showed that TGF-β1 was obviously reduced in SK-BR-3 and HCC1806 cells at both low (even 10 µg/ml) and high concentrations of LAE (Fig. 2a). To further determine whether TGF-β1 signal was required for LAE-inhibited cell migration, 10 ng/ml TGF-β1 growth factor with 100 µg/ml LAE was used to treat SK-BR-3, MDA-MB-231 and HCC1806 cells. LAE-inhibited cell migration was significantly restored by the addition of TGF-β1 growth factor, as revealed by wound healing assays (Fig. 2b). Consistent with literatures, TGF-β1 growth factor changed cells morphology from epithelial to mesenchymal transition (EMT), which was suppressed by LAE supplement (Fig. 2c). Thus, these observations demonstrated that TGF-β1 signal is involved in LAE-inhibited cell migration likely via inhibition of EMT.

### SMAD3 and Erk1/2 phosphorylation are inhibited

**Fig. 3:**
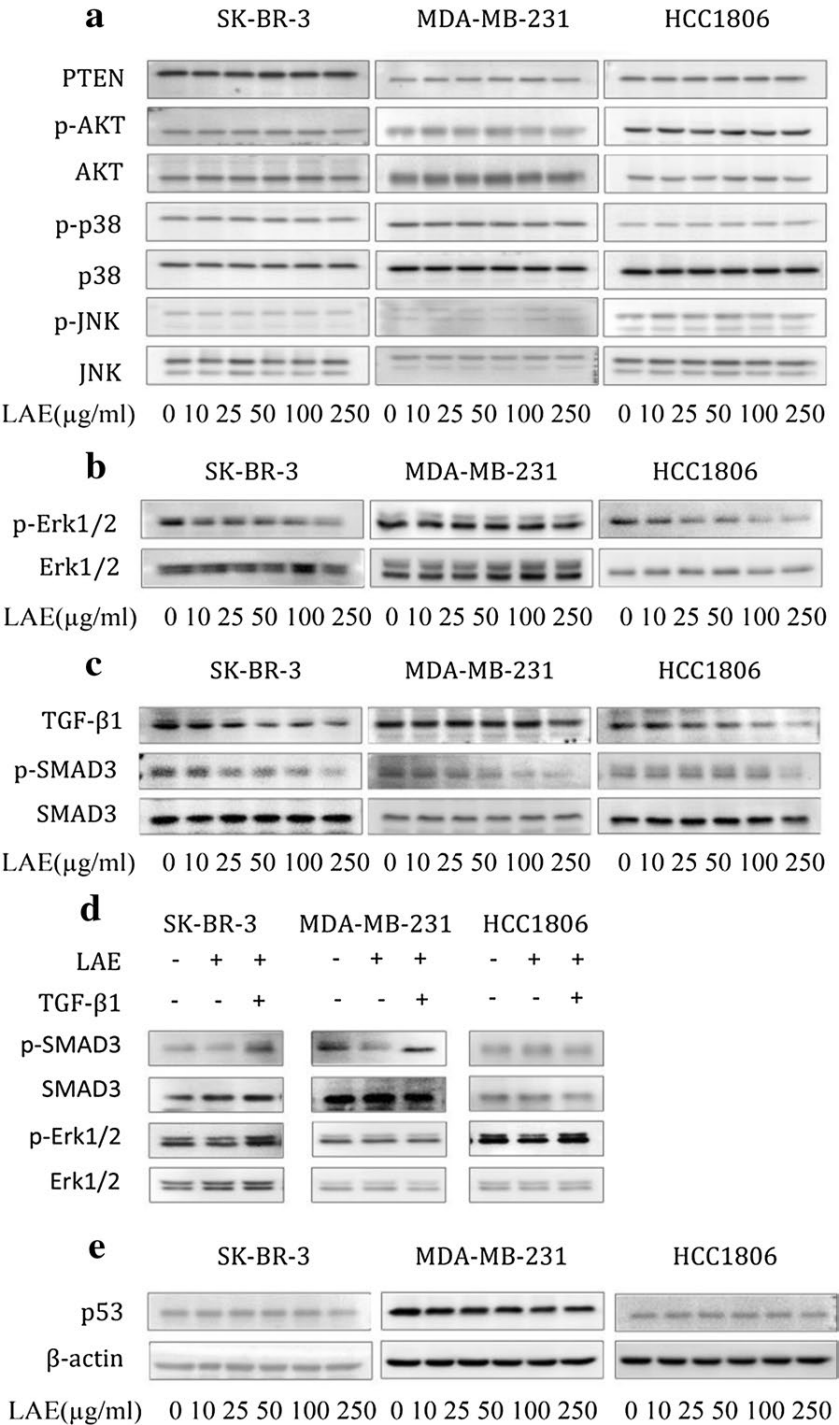
SMAD3 and Erk1/2 phosphorylation levels. **a** PTEN, p-Akt, Akt, p-p38, p38, p-JNK and JNK were determined by immunoblotting under different concentrations of LAE (0, 10, 25, 50, 100, 250 µg/ml). **b** p-Erk1/2 and Erk1/2 were examined by immunoblotting; **c** p-SMAD3 and SMAD3 were examined by immunoblotting; **d** p-SMAD3 and p-Erk1/2 proteins derived from cells treated with 100 µg/ml LAE alone or combined with TGF-β1 (10 ng/ml). **e** p53 protein level was examined by immunoblotting

To figure out the signaling pathway involved in LAE-inhibited cell migration, we examined the canonical TGF-β1/SMAD3 signaling pathway and the non-canonical TGF-β1/PTEN/AKT and MAPKs signaling pathways [28, 29]. Immunoblotting analysis showed that AKT phosphorylation and PTEN levels were unchanged in three subtypes of breast cancer cells with LAE supplement (Fig. 3a). Similar p38 and JNK phosphorylation levels were observed in cells with and without LAE supplement (Fig. 3a). However, the phosphorylated Erk1/2 level was significantly reduced in SK-BR-3 and HCC1806 cells even at a low LAE supplement (10 µg/ml), and mildly reduced in MDA-MB-231 cells (Fig. 3b). Interestingly, with LAE supplement, the phosphorylated-SMAD3 level was significantly reduced in SK-BR-3 and MDA-MB-231 cells, but not in HCC1806 cells (Fig. 3c). Moreover, TGF-β1 supplement significantly restored Erk1/2 and SMAD3 phosphorylation levels individually or simultaneously which is inhibited by LAE supplement in three subtypes of breast cancer cells (Fig. 3d).

Mutant p53 mediates the TGF-β1 signaling pathway via Erk1/2 as well as SMAD3 signals, and the p53 in all of the three subtypes of breast cancer cells are mutant [30–33]. We examined the mutant p53 protein level in three subtypes of breast cancer cells with LAE supplement. Although p53 level was similar in SK-BR-3 and HCC1806 cells with and without LAE supplement, immunoblotting results showed that p53 protein level was significantly decreased in MDA-MB-231 cells with LAE supplement in a dose dependent manner (Fig. 3e). Together, these results demonstrated that LAE inhibits the cell migration of ER^−^ breast cancer cells likely via suppressing the phosphorylation of TGF-β1/Erk1/2 as well as TGF-β1/SMAD3 signals.

### LAE inhibits TGF-β1-related cell migration via downregulating hydrogen peroxide

**Fig. 4:**
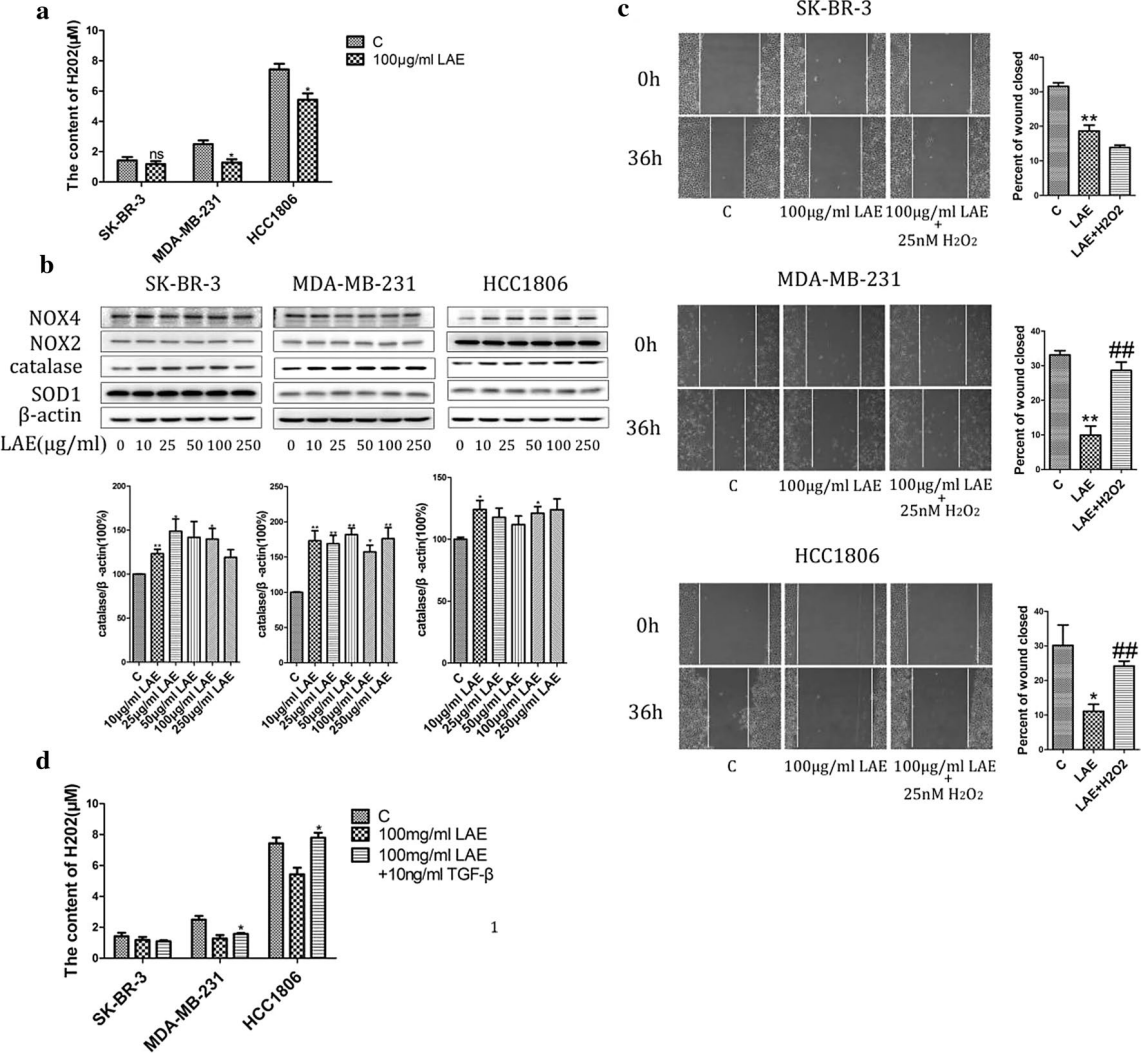
Hydrogen peroxide level in LAE-inhibited cell migration. **a** The content of intracellular H_2_O_2_ was determined after treating with 100 µg/ml LAE for 48 h in SK-BR-3, MDA-MB-231 and HCC1806. **b** NOX4, NOX2, catalase and SOD1 proteins levels were examined by immunoblotting with different concentrations of LAE (0, 10, 25, 50, 100, 250 µg/ml). **c** Wound healing assays after treating with LAE alone or combined with 25 nM H_2_O_2_ for 36 h (× 100 magnification). **d** The content of intracellular H_2_O_2_ was determined after treating with 100 µg/ml LAE alone or combined with TGF-β1 (10 ng/ml) for 48 h in SK-BR-3, MDA-MB-231 and HCC1806. Values represent the mean ± SD (n = 3)

Reactive oxygen species (ROS), especially hydrogen peroxide (H_2_O_2_), are associated with tumor metastasis [34–36]. TGF-β-related signals promote hydrogen peroxide production in several types of cells [37–39]. H_2_O_2_ enhances TGF-β-mediated EMT via SMAD and MEK/Erk signaling pathways [40]. To determine if intracellular H_2_O_2_ participate in the TGF-β1-related cell migration inhibition, we first measured H_2_O_2_ level in cells with LAE supplement for 48 h. With LAE supplement, intracellular H_2_O_2_ level was significantly downregulated in MDA-MB-231 and HCC1806 cells, but not in SK-BR-3 (Fig. 4a). Consistently, H_2_O_2_ scavenger catalase protein level was significantly increased even at a low concentration of LAE supplement (10 µg/ml) (Fig. 4b). However, SOD1, NOX2 and NOX4 protein levels, responsible for the production of large amounts of ROS, were similar in cells with or without LAE supplement. Thus, LAE supplement reduced H_2_O_2_ production in MDA-MB-231 and HCC1806 cells, likely via enhancing the catalase level.

To determine if downregulated H_2_O_2_ level caused LAE-induced inhibition of breast cancer cell migration, we added 25 nM H_2_O_2_ with 100 µg/ml LAE supplement to treat SK-BR-3, MDA-MB-231 and HCC1806 cells. Wound healing distance results showed that the addition of H_2_O_2_ significantly restored LAE-inhibited cell migration in MDA-MB-231 and HCC1806 cells (Fig. 4c). However, SK-BR-3 cells displayed similar cell migration with or without H_2_O_2_ supplement, suggesting other signals involved in LAE-inhibited cell migration. Interestingly, elevated intracellular H_2_O_2_ level was observed in MDA-MB-231 and HCC1806 cells with 10 ng/ml TGF-β1 and 100 µg/ml LAE compared to cells with single LAE supplement, but not in SK-BR-3 cells (Fig. 4d). These findings indicated that LAE inhibits TGF-β1-mediated cell migration via downregulating hydrogen peroxide in MDA-MB-231 and HCC1806 cells.

### LAE inhibits tumor metastasis

**Fig. 5:**
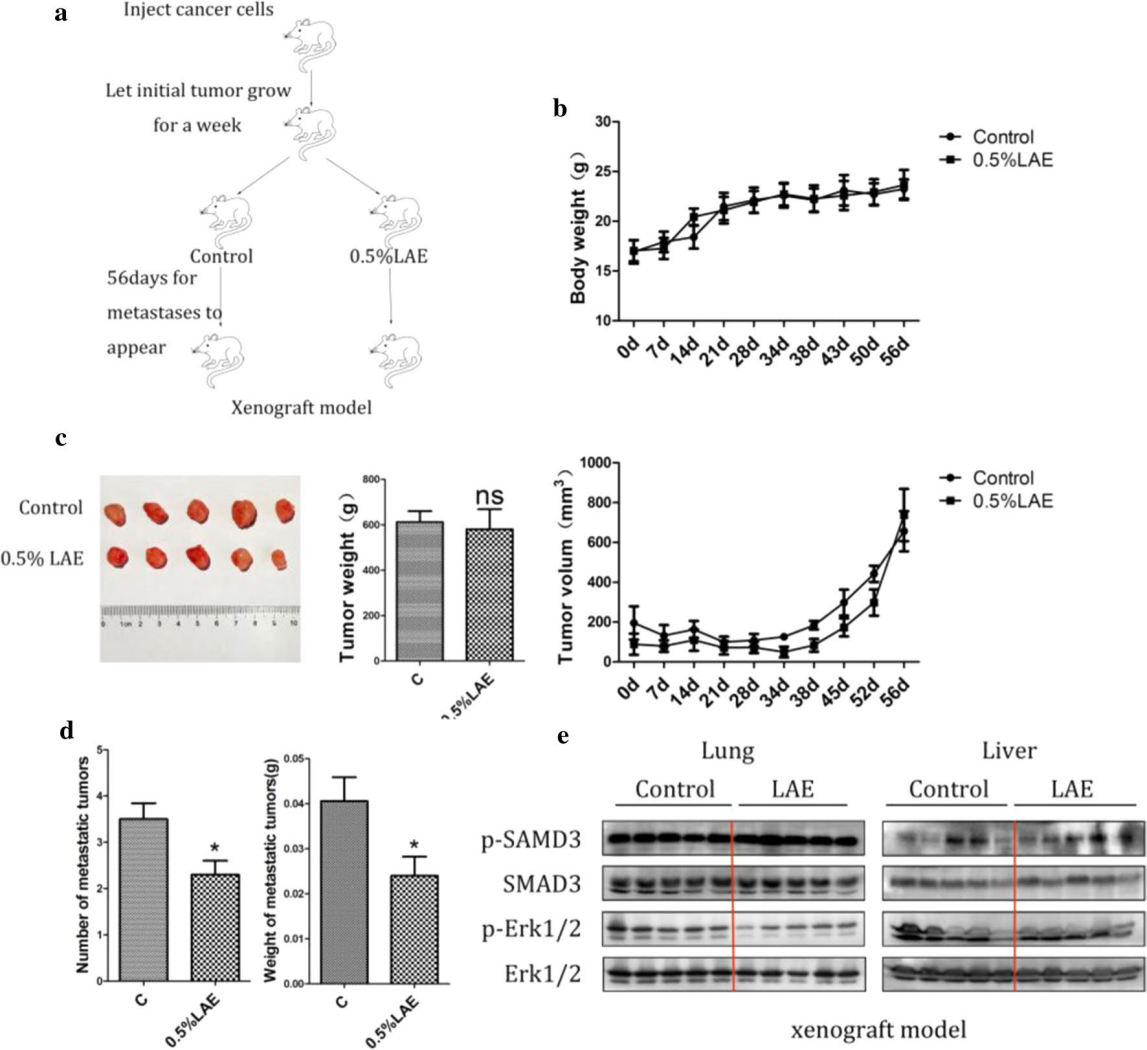
LAE effect on tumor metastasis in xenograft model. a Flowchart of animal experiments. MDA-MB-231 (5 × 10^7^) were subcutaneously injected in the right flank of mice. After 1 week of injection, the tumor bearing mice were randomly subdivided into 2 groups: Control; 0.5% LAE. **b** Growth curves of body weight in xenograft model. **c** Upper panel: Representative tumors and weight of tumors. Lower panel: Growth curves of tumor volume in xenograft model. **d** The numbers and weights of new metastatic tumors derived from the primary tumor. **e** p-SMAD3 and p-Erk1/2 proteins levels were examined by immunoblotting in lung and liver tissues (xenograft model)

To investigate the inhibitory effects of LAE on metastasis in vivo, we used subcutaneous xenograft nude mice models to examine tumor metastasis. The schedule of experiments was showed in Fig. 5a. To exclude the toxicity of LAE in vivo, xenograft nude female mice were fed with 0.5% LAE for 56 days and body weight of mice was monitored. Body weight in mice with and without LAE supplement was similar (Fig. 5b), indicating that LAE supplement did not cause systemic toxicity. Consistent with Fig. 1, LAE supplement did not alter the growth of in-situ tumor compared to controls (Fig. 5c). However, the number and weight of new metastatic tumors derived from the primary tumor were significantly decreased in mice with LAE supplement compared to controls (Fig. 5d). Immunoblotting results showed that LAE did not inhibit the phosphorylation of Erk1/2 and SMAD3 in liver. However, LAE supplement significantly downregulated the phosphorylation of Erk1/2 but not SMAD3 in lung (Fig. 5e). These data indicated that the effect of LAE on SMAD3 and Erk1/2 is dependent on tissue environment or extracellular signal stimulation.

**Fig. 6:**
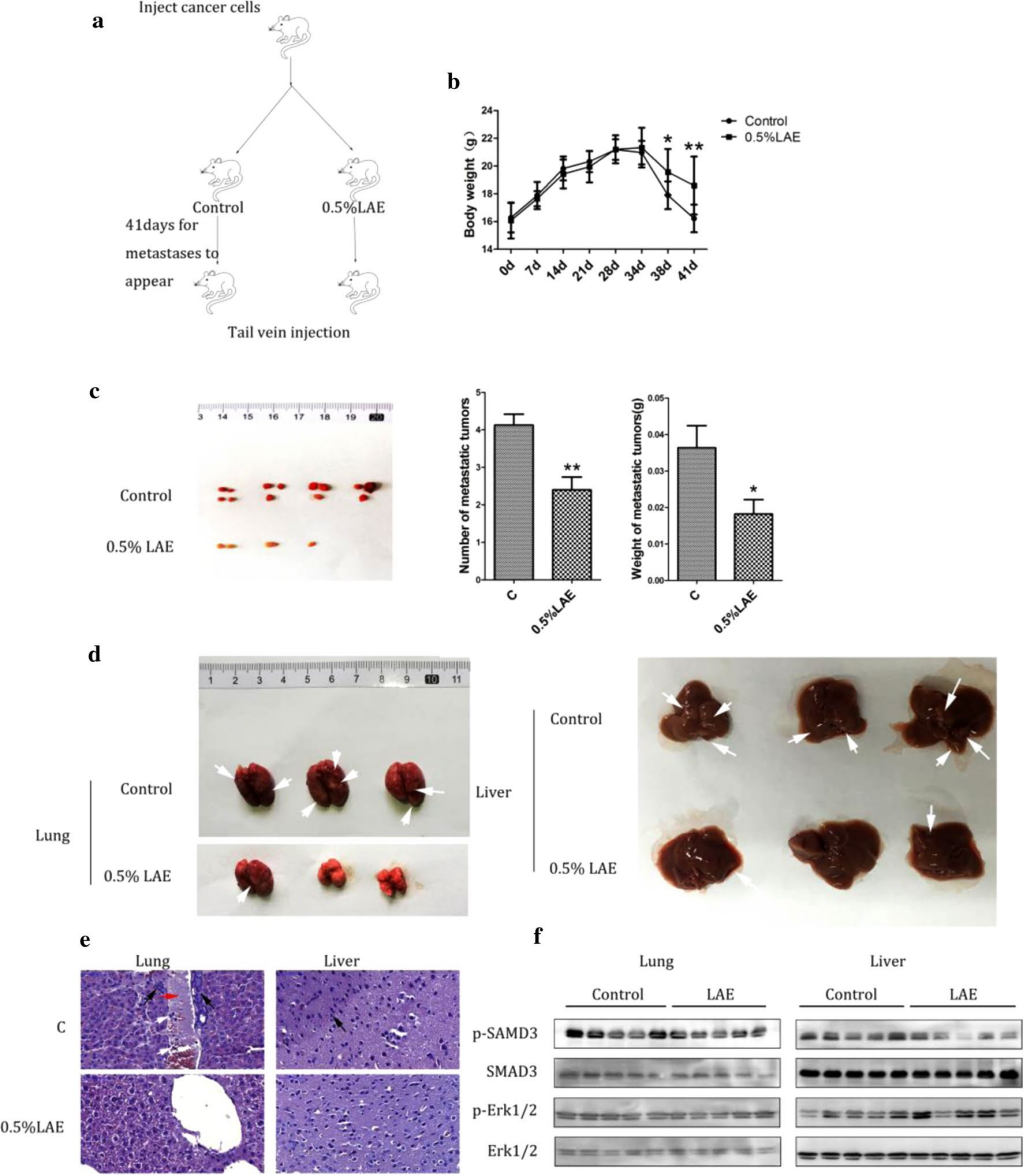
LAE effect on tumor metastasis in tail vein injection model. **a** Flowchart of animal experiments. MDA-MB-231 (5 × 10^7^) were intravenous injected in nude mouse. After 1 week of injection, the tumor bearing mice were randomly subdivided into 2 groups: Control; 0.5% LAE. **b** Growth curves of body weight in intravenous injection nude mouse model. **c** Left panel: Representative new metastatic tumors which formed after injecting the MDA-MB-231 breast cancer cells into the vein of mice; Middle and right panels: The numbers and weights of new metastatic tumors. **d** Representative lung and liver tissues of intravenous injection nude mice. White arrows represent the metastatic tumor nodules. **e** Metastatic tumor nodules in the lung and liver tissues were examined by H&E staining (White arrow: Cancer cells invade the lumen of a large blood vessel. Black arrow: Metastatic tumor nodules substitute a segment of the vascular wall or exist in the liver tissues. Red arrow: inflammatory infiltration in blood vessel). **f** p-SMAD3 and p-Erk1/2 levels were examined by immunoblotting in lung and liver tissues (tail vein injection model). Values represent the mean ± SD (n = 3)

Next, we used intravenous injection nude mice models to investigate the anti-metastatic role of LAE. The experiments schedule was showed in Fig. 6a. Strikingly, LAE supplement significantly suppressed mice body weight loss induced by tumor (Fig. 6b), which indicated that LAE may improve the weight loss in mice with advanced breast cancer. And the number and weight of new formed metastatic tumors were significantly decreased in mice with LAE supplement (Fig. 6c). Interestingly, we found less lung congestion in mice with LAE supplement than that of control animals (Fig. 6d). The number and size of metastatic tumor nodules in lung and liver was decreased significantly. H&E staining revealed inflammatory infiltration and vascular invasion in lung of mice, while LAE supplement improved inflammatory infiltration and vascular invasion in lung of mice (Fig. 6e). In liver, LAE supplement significantly decreased the number of large metastatic tumors. Mice with LAE supplement showed significantly downregulated the phosphorylation of SMAD3 in lung and liver, but not the phosphorylation of Erk1/2 (Fig. 6f). Taken together, these data suggest that LAE inhibited tumor metastasis, at least in part, via suppressing the phosphorylation of SMAD3 and Erk1/2.

### Extracellular matrix signal are associated with LAE-suppressed cell migration

To further explore the molecular mechanism of LAE inhibiting cell migration, we performed RNA-sequence to analysis gene expression profiles in SK-BR-3, HCC1806 and MDA-MB-231 cells after LAE treatment. All the differentially expressed genes were analyzed by (Gene Ontology) GO method in DAVID with the standard of *P* < 0.001 and FDR < 1. Results showed that extracellular matrix (ECM) organization, oxidation–reduction process, negative regulation of cell migration and cell adhesion were significantly enriched by LAE supplement in MDA-MB-231 cells (Additional file 1: Fig. S2a), and oxidation–reduction process, extracellular matrix organization, cell–cell adhesion and cell migration were significantly enhanced by LAE supplement in SK-BR-3 cells (Additional file 1: Fig. S2b), while extracellular region part and extracellular matrix part were significantly changed by LAE supplement in HCC1806 cells (Additional file 1: Fig. S2c). Furthermore, Kyoto Encyclopedia of Genes and Genomes (KEGG) pathway analysis found that pathways involved in ECM-receptor interaction and focal adhesion as well as their related genes were significantly enriched (Additional file 1: Fig. S2d–f) in SK-BR-3, HCC1806 and MDA-MB-231 cells. Notably, the ECM components and cell adhesion could be induced by the TGF-β1 and FAK signaling pathways [41–44]. Taken together, our results demonstrated that LAE-suppressed cell migration are associated with multiple signaling pathways in different cell lines, likely via extracellular matrix signal.

## Discussion

Breast cancer, especially the ER^−^ breast cancer, is harmful to the health of women because of its metastasis, which lead to more than 90% of breast cancer deaths [45, 46]. Tumor metastasis is the dissemination of cancer cells from the initial site to distant organs, including epithelial-mesenchymal transition (EMT), cell migration, local tissue invasion, intravasation, extravasation, and metastatic niche formation. EMT endues cells not only migration ability but invasive properties and stem-like cells functions [47–49]. In the present study, we demonstrated that LAE inhibited EMT and cell migration via Erk1/2 and SMAD3 and reducing H_2_O_2_ production in ER^−^ breast cancer. Our findings provided a new possible therapeutic approach for ER^−^ breast cancer.

Although the anti-bacterial, anti-oxidant, and anti-obesity effects of lotus extracts have been known for many years [22, 50], little is known about the activity of LAE on cancer cell migration. We demonstrated that LAE had the potential inhibitory activity for cell migration and EMT in vitro and in vivo. However, few components of lotus leaf with the effects of anticancer have been revealed. Previous studies showed that nuciferine, a major bioactive component of lotus, inhibits the growth of cancer cells and breast cancer-associated bone loss [23, 51]. However, our results showed that nuciferine was not involved in LAE-inhibited migration. Thus, further experiments are required for identification of bioactive compound in LAE of inhibiting cell migration and metastasis.

In this study, the percent of wound closed in HCC 1806 control cells showed the large variance, but the inhibitory effects of LAE on cell migration are still remained. Different cell density might lead to this variance. As already reported, the more densely populated samples conferred a faster wound closure simply due to the more motile and higher concentration of paracrine signaling at higher cell seeding densities [52]. In addition, the variation may result from the discrepant composition in different batches extracts. Furthermore, with the extension of time, the antioxidant components such as flavonoids are prone to be oxidized. These discrepancies might affect the inhibitory effects of LAE on cell migration due to the potential variation in LAE composition. It should be the focus of our future research to determine which factor(s) specifically enhance the inhibitory activity of LAE on cell migration and metastasis.

Many signaling pathways are associated with the cancer cell migration. TGF-β1 modulates the EMT process by the canonical TGF-β1/SMAD3 signaling pathway and the non-canonical TGF-β1/PTEN/AKT and MAPKs signaling pathways. Our results indicated that LAE inhibited TGF-β1 signaling pathway via Erk1/2 and SMAD3 in SK-BR-3, MDA-MB-231 and HCC1806 breast cancer cells. Furthermore, many cytokines and receptors involving metastasis are different in the ER^−^ breast cancer cells [53]. These differences among the three cell lines with LAE treatment may represent different effects of TGF-β pathways in basal-like breast cells (HCC 1806), HER2+ (SK-BR-3) and mesenchymal-like breast cells (MDA-MB-231).

To investigate the inhibitory effects of LAE on metastasis in vivo, we used two metastasis mice models. The results in vivo showed that LAE inhibited tumor metastasis via suppressing the phosphorylation of SMAD3 and Erk1/2. The different effect of LAE on SMAD3 and Erk1/2 in mice models might be explained by the diversity of the extracellular matrix compounds and cell microenvironment between the in vitro and in vivo studies, which is revealed by the results of GSEA. Consistently, Literatures have demonstrated that the SMAD3 and Erk pathways are also regulated by other factors in the tumor environment including steroid hormones and epidermal growth factor [54, 55]. Furthermore, several studies reported that SMAD3 increases the adhesive ability of hepatocellular carcinoma (HCC) cells by the form of exosome, which promotes the lung metastasis [56]. These might explain why LAE treatment significantly changed the ECM and cell–cell adhesive signaling pathways, which were regulated by the TGF-β1 and FAK signaling pathway. Thus, ECM and FAK might be other targets to inhibit the cell migration and metastasis induced by LAE.

In our study, the subcutaneous xenograft and tail vein injection models were used to mimic the early and advanced stage of breast cancer. Interestingly, results showed that LAE has different effects on Erk1/2 and SMAD3 activation in the two models. The differences might be explained by the different tumor stage. MAPK pathways, including p38 and Erk pathways are sensitive to the tumor microenvironment [57]. The activated Erk is a master regulator for the tumor progression. In the early stage of tumor metastasis, many processes regulated by Erk, such as angiogenesis, are earlier than the SMAD3 pathway [58]. Above all, despite of the differences among the three cell lines and between the in vitro and in vivo studies, our findings suggested that LAE inhibits the cell migration and metastasis of ER^−^ breast cancer.

Literatures have demonstrated that activated Erk1/2 stimulates the metastasis in cancers by targeting Snail, Slug and matrix metalloproteinases (MMPs) [59]. However, in the present work, we demonstrated that LAE suppressed the activation of Erk1/2 and SMAD3 as well as reduced the intracellular H_2_O_2_ levels, which were regulated by TGF-β1 signaling pathway in MDA-MB-231 and HCC1806 cells, but not in SK-BR-3 cells. Exogenous H_2_O_2_ significantly restored LAE-inhibited cell migration in MDA-MB-231 and HCC1806 cells, but not SK-BR-3, revealed that H_2_O_2_ was not involved in LAE-inhibited cell migration in SK-BR-3 cells. Interestingly, although LAE treatment significantly reduced the phosphorylation levels of Erk1/2 and SMAD3, the expression of TGF-β1 protein which regulated them both did not change significantly in MDA-MB-231 cells. This might be explained by the significantly decreased expression of mutant p53 in MDA-MB-231 cells because of the essential role of mutant p53 in mediating the TGF-β1 signaling pathway [30]. Taken together, our findings strongly suggested that LAE inhibit EMT by primarily targeting the TGF-β1 pathway, and the underlying mechanism of LAE-suppressed cell migration is differently within the three cell lines.

## Conclusion

In this study, our data demonstrated that LAE significantly inhibits the ER^−^ breast cancer cell migration and metastasis via the TGF-β1/Erk1/2 and TGF-β1/SMAD3 signaling pathways, accompanied by reduced intracellular H_2_O_2_ level in ER^−^ breast cancer cells. Extracellular matrix signaling pathways and FAK might be other possible pathways to inhibit the cell migration induced by LAE. These results suggested that LAE has the potential to figure out novel components to develop drugs for the treatment of ER^−^ breast cancer clinically.

## Supplementary Information

**Additional file 1: Fig. S1**. Wnt and autophagy signaling pathways. **Fig. S2**. Gene expression profiles of cells with LAE supplement by RNA-sequence. **Fig. S3**. Nuciferine effect on the inhibition of cell migration by LAE.

## Data Availability

The datasets from the present study are available from the corresponding author upon request.

## Abbreviations

TGF-β1: Transforming growth factor-β1
ER+/PR+: Estrogen receptor positive/progesterone receptor positive
HER2+: Human epidermal growth factor receptor positive
TNBC: Triple negative
LAE: Lotus leaf alcoholic extract
CQ: Chloroquine diphosphate
H&E: Hematoxylin and eosin
ROS: Reactive oxygen species
ECM: Extracellular matrix
