# Supplementary material for: Lotus leaf extract inhibits ER− breast cancer cell migration and metastasis
Source: Nutr Metab (Lond). 2021 Feb 18;18:20. doi: 10.1186/s12986-021-00549-0 (PMC7891157; doi:10.1186/s12986-021-00549-0)
Supplement: Supplementary file 1 — Additional file 1: Fig. S1. Wnt and autophagy signaling pathways. Fig. S2. Gene expression profiles of cells with LAE supplement by RNA-sequence. Fig. S3. Nuciferine effect on the inhibition of cell migration by LAE. [file 12986_2021_549_MOESM1_ESM.doc]

**
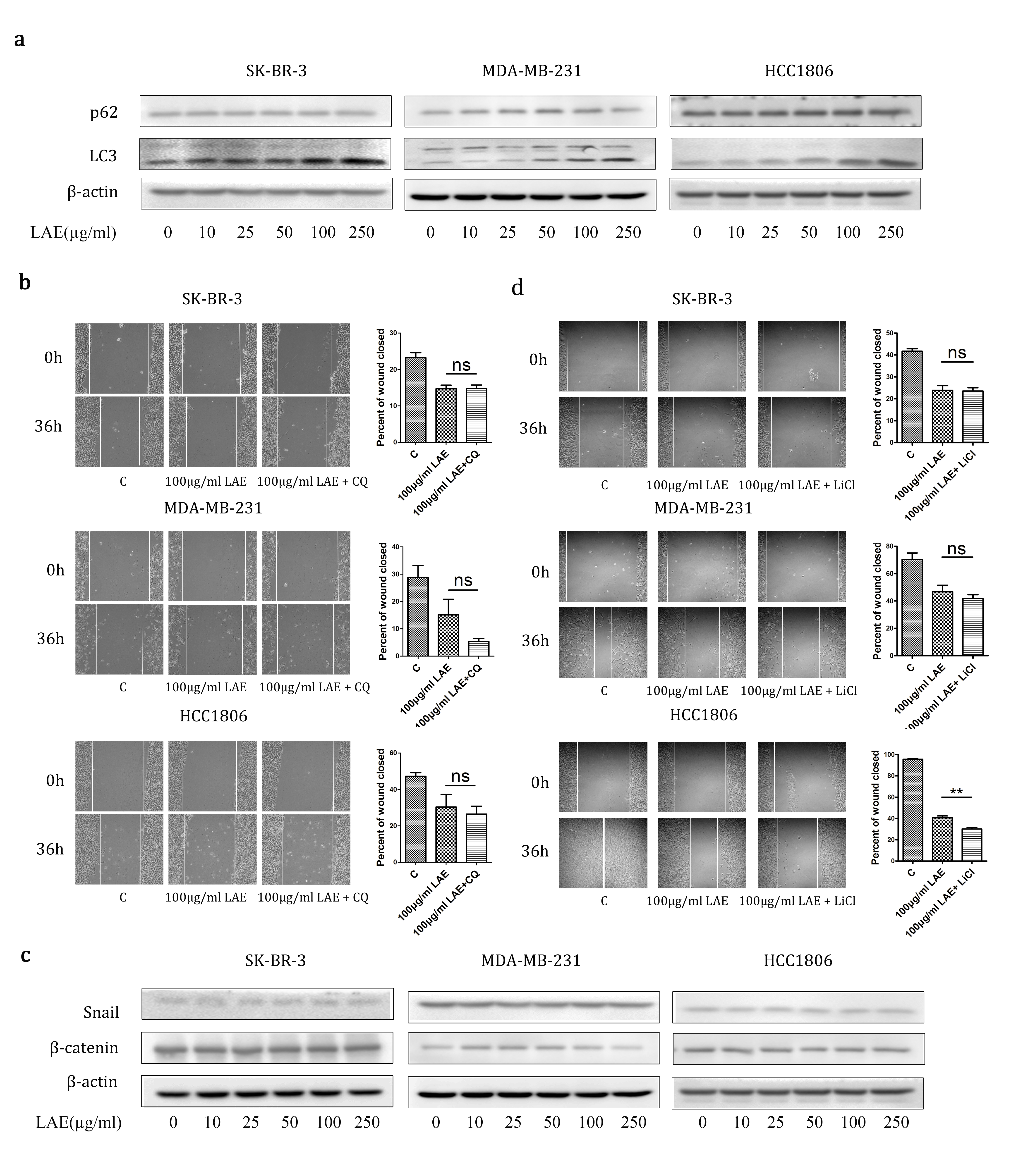
**

**Supplementary Fig. 1** Wnt and autophagy signaling pathways. **a** p62 and LC3 proteins levels were determined by immunoblotting with indicated concentrations of LAE (0,10,25,50,100,250µg/ml). **b** Wound healing assays with LAE alone and combined with CQ (10mg/ml); **c** β-catenin and Snail proteins levels were determined by immunoblotting with indicated concentrations of LAE (0,10,25,50,100,250µg/ml). **d** Wound healing assays with LAE alone and combined with LiCl (20mM) for 36h.


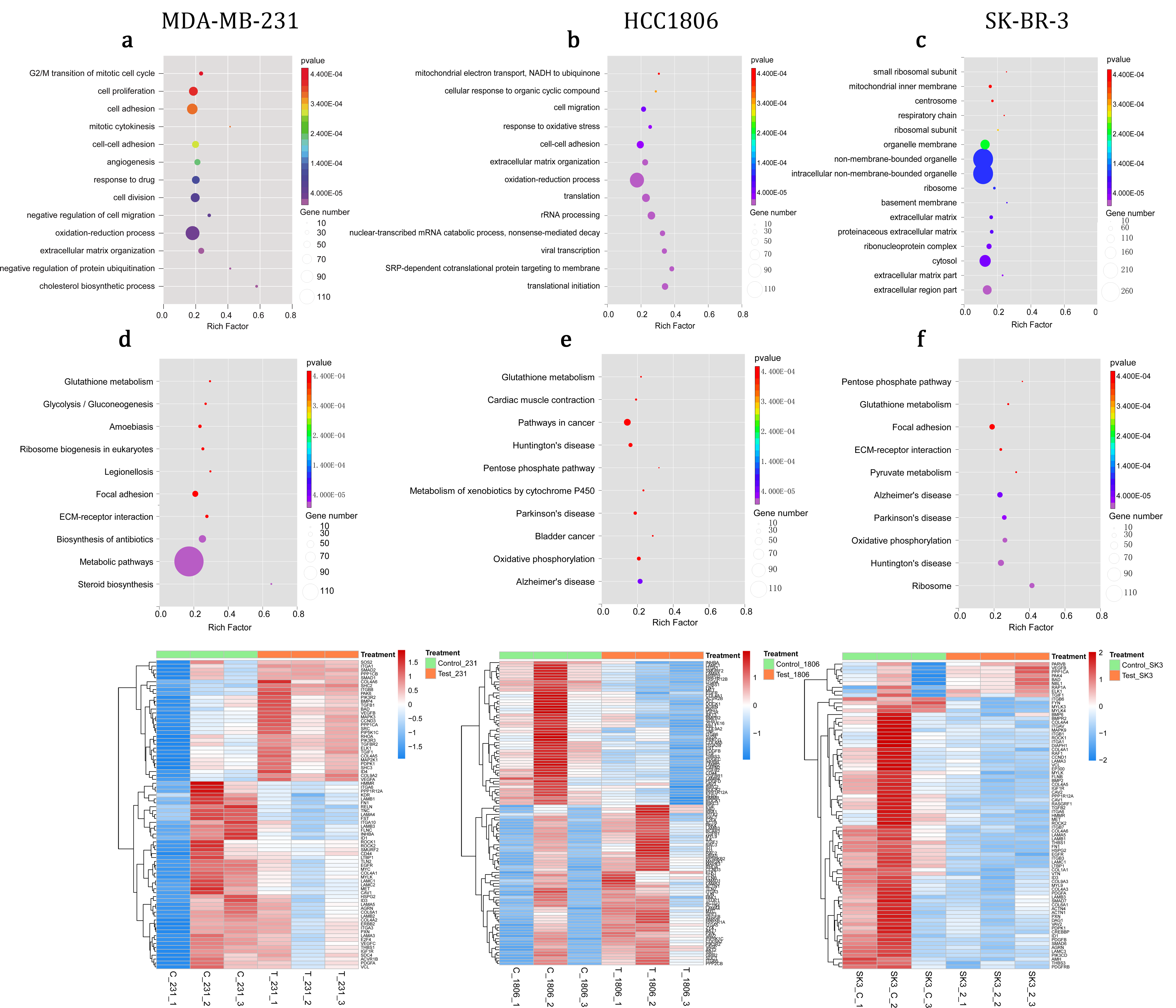


**Supplementary Fig. 2** Gene expression profiles of cells with LAE supplement by RNA-sequence. **a-c** Significantly enriched GO terms for biological process in MDA-MB-231 and SK-BR-3 and cellular component in HCC1806 after treating with LAE. **d-f** KEGG analysis and gene expression pattern analysis about ECM-receptor interaction and focal adhesion in MDA-MB-231, HCC1806, SK-BR-3 cell lines.


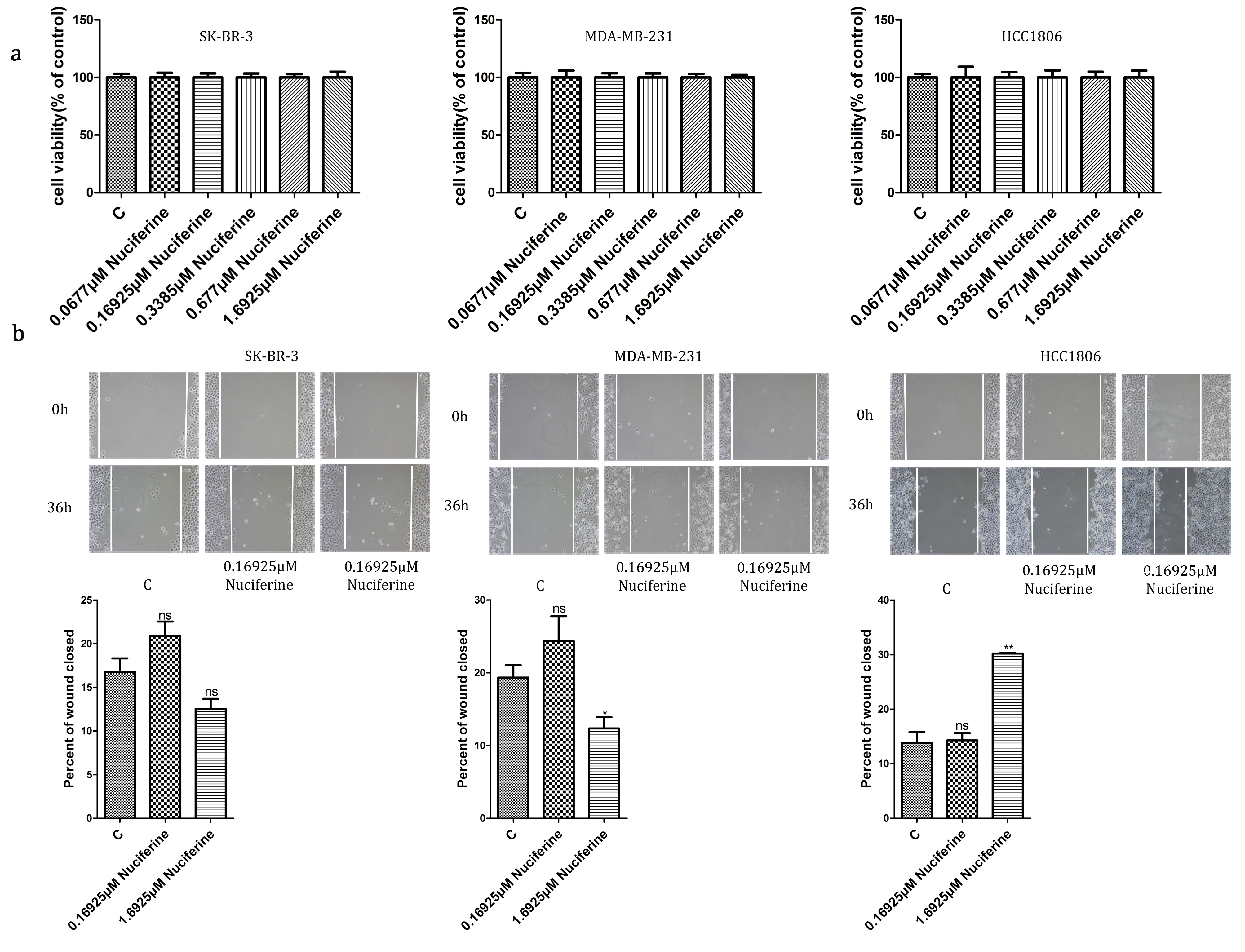


**Supplementary Fig. 3** Nuciferine effect on the inhibition of cell migration by LAE. **a** The viability of nuciferine-treated cells was determined by CCK8 assays. **b** Wound healing assays after treating with nuciferine for 36h (x100 magnification). Values represent the mean ± SD (n = 3).
